# Effective management of 5-fluorouracil-induced coronary vasospasm allowing chemotherapy continuation: a single-center observational study

**DOI:** 10.1186/s40959-026-00526-7

**Published:** 2026-06-16

**Authors:** Kinga Kolossváry, Krisztina Heltai, Borbála Székely, Gábor Rubovszky

**Affiliations:** 1 Department of Thoracic and Abdominal Oncology and Clinical Pharmacology, ”Chemotherapy B” and the National Tumor Biology Laboratory, National Institute of Oncology, Ráth György U. 7-9, 1122 Budapest, Hungary; 2 National Institute of Oncology, Outpatient Cardiology Clinic, Budapest, Hungary

**Keywords:** Cardio-oncology, 5-Fluorouracil, Cardiotoxicity, Coronary vasospasm, Calcium channel blocker, Nitrates, Antianginal treatment

## Abstract

**Background:**

5-fluorouracil (5-FU) is a widely used chemotherapy agent in gastrointestinal (GI) malignancies, but it can cause cardiotoxicity, most commonly manifesting as chest pain due to coronary vasospasm, often mimicking acute coronary syndrome. Adequate management of 5-FU-associated vasospasm is critical to ensure the necessary oncological treatment can be safely continued.

**Objectives:**

The aim of this study was the retrospective analysis of cases of chest pain associated with intravenous 5-FU treatment in GI cancer patients at a single institution, and to report the experiences with antianginal treatment, specifically nitrates and calcium channel blockers, administered in these cases.

**Methods:**

This was a retrospective, single-center, observational cohort study. We analyzed data of GI cancer patients treated with intravenous 5-FU between March 2024 and August 2025 who experienced chest pain during treatment. Following the institutional protocol, the chemotherapy was transiently suspended, and treatment was re-introduced under combined oral or intravenous nitrate and calcium channel blocker protection.

**Results:**

Out of 835 patients receiving iv 5-FU, 25 (2.9%) developed chest pain, typically after the second or third cycle of chemotherapy. Three patients presented with ST-elevation on ECG, but none of them showed significant coronary stenosis on imaging. With appropriate cardiological treatment, all 25 patients achieved symptom-free oncological treatment continuation: 19 patients with combined nitrate and calcium channel blocker therapy, 6 with monotherapy, and one of these patients required intravenous treatment under ICU monitoring.

**Conclusions:**

Coronary vasospasm associated with intravenous 5-FU is a rare but clinically significant complication. Our findings, in line with the literature, suggest that the introduction of antianginal therapy successfully allowed the safe rechallenge and continuation of oncological therapy in all affected patients, which may prevent unnecessary discontinuation of life-prolonging chemotherapy.

**Trial registration:**

Retrospectively registered (Approval No. OOI/Ált11147-1/2025).

## Background

Gastrointestinal malignancies—including cancers of the esophagus, stomach, small intestine, pancreas, and colorectum—account for a substantial proportion of cancer-related mortality worldwide [1]. Despite improvements in survival resulting from advances in early detection and oncologic therapies, the cardiovascular adverse effects of chemotherapy remain a significant clinical challenge. Fluoropyrimidines are currently the third most commonly used class of chemotherapeutic agents in solid tumors, particularly in gastrointestinal, breast, and head and neck cancers [1, 2]. They are integral components of standard chemotherapy regimens such as FOLFOX and FOLFIRI and are administered either as bolus injections or continuous intravenous infusions or orally, like capecitabine. Although their oncologic efficacy is well established, fluoropyrimidines are associated with a broad spectrum of adverse effects [3].

Common toxicities include gastrointestinal symptoms (e.g., diarrhoea, nausea), bone marrow suppression, and hand-foot syndrome [4]. Increasing attention has been directed toward the less frequent but potentially severe cardiotoxic effects of 5-fluorouracil (5-FU), which may necessitate treatment interruption. Coronary vasospasm represents the most characteristic manifestation of 5-FU-associated cardiotoxicity and typically presents with angina-like chest pain. Less frequently, arrhythmias, pericarditis, myocarditis, heart failure, and sudden cardiac death have been reported [4].

5-FU–associated coronary vasospasm most commonly occurs during the infusion or within several days thereafter and may present with acute chest pain, electrocardiographic abnormalities (including ST-segment elevation or depression), and/or elevation of cardiac biomarkers. Clinically, this presentation may closely mimic acute coronary syndrome (ACS) [1, 5].

The underlying mechanisms of 5-FU–induced cardiotoxicity are multifactorial and incompletely understood. Vasospasm may result from endothelium-dependent mechanisms involving direct endothelial injury or from endothelium-independent pathways related to vascular smooth muscle dysfunction [3, 6]. Oxidative stress has also been implicated in the pathogenesis of vasospasm [3]. Endothelial dysfunction induced by 5-FU may lead to a paradoxical vasoconstrictive response to acetylcholine, further predisposing patients to ischemic events [3, 6–8].

Early recognition and appropriate management are essential, particularly in patients for whom continuation of oncologic therapy is critical. Although 5-FU-associated vasospasm carries a significant cardiovascular risk, chemotherapy may be safely continued in selected cases following comprehensive cardiologic evaluation and, when indicated, coronary angiography, in conjunction with nitrate and calcium channel blocker antianginal therapy [6]. Close cardiovascular monitoring is mandatory, as recurrence of symptoms is common [4, 9].

Evidence consistently demonstrates that 5-FU cardiotoxicity is schedule-dependent, with continuous infusion carrying a significantly higher risk of coronary vasospasm than bolus administration. Landmark studies report incidence rates of 6–6.3% for continuous infusion compared to 2.1–2.2% for bolus regimens [10–12]. This elevated risk, which also extends to oral capecitabine [12], likely stems from prolonged endothelial exposure and a sustained imbalance between vasoconstrictors and vasodilators. Furthermore, the addition of leucovorin may potentiate this effect [11]. Despite their date of publication, these data remain definitive as the fundamental pharmacokinetics and scheduling of fluoropyrimidines in oncology protocols have remained unchanged [13].

Advances in the rapidly evolving field of cardio-oncology have led to improved recognition and management of cardiovascular complications associated with cancer therapies [14]. In Hungary, the management of 5-FU-induced anginal symptoms dates back to the early 2000 s, when intravenous calcium channel blockers and nitrates were successfully administered concomitantly with 5-FU-based chemotherapy at the National Institute of Cardiology.

### Objectives

The objective of the present study was to perform a retrospective analysis of patients with GI malignancies who developed chest pain during intravenous 5-fluorouracil therapy at the National Institute of Oncology, with particular focus on the use of nitrate and calcium channel blocker treatment in symptomatic cases.

## Methods

### Ethical approval

The study was conducted in accordance with the principles of the Declaration of Helsinki. Ethical approval was granted by the Regional Research Ethics Committee (Approval No. OOI/Ált11147-1/2025). Owing to the retrospective and anonymized nature of the data analysis, the requirement for individual informed consent was waived by the Committee.

### Study design and setting

This study was a protocol-based, non-interventional, retrospective, single-center observational cohort study. It was conducted at the Day Care Unit of the National Institute of Oncology. The study period extended from March 1, 2024, to August 1, 2025.

The primary objective was to retrospectively evaluate the effectiveness and safety of the institution's standard cardio-oncology antianginal protocol implemented for the management of coronary vasospasm—related chest pain occurring during intravenous 5-fluorouracil (5-FU)—based chemotherapy. Given the retrospective nature of the study, detailed implementation of specific protocol elements (drug selection, dosing, and timing) is presented in the Results section based on analysis of the collected clinical data.

### Data sources

The total number of patients receiving intravenous 5-FU therapy during the study period was determined using institutional pharmacy records, which accurately document dispensed and administered chemotherapeutic agents. Subsequent data collection was performed using the institution's electronic hospital information system (MedWorks). Patients who developed chest pain during chemotherapy were identified through a targeted keyword search for the long-acting nitrate (Olicard) preparation routinely used according to institutional protocol. Clinical data required for analysis—including demographic characteristics, oncologic diagnosis, chemotherapy regimen, timing of chest pain onset, electrocardiographic abnormalities, administered antianginal therapies, and outcomes of chemotherapy rechallenge—were recorded in an encrypted, password-protected Microsoft Excel database.

### Case definition

Presumed coronary vasospasm was defined as the occurrence of typical angina-like chest pain during 5-FU infusion or between treatment cycles, in the absence of another evident cardiac cause, regardless of whether transient electrocardiographic changes (ST-segment or T-wave abnormalities) were recorded.

### Inclusion and exclusion criteria

Patient selection followed a predefined protocol. During the study period, a total of 835 patients with gastrointestinal malignancies received intravenous 5-FU therapy.

#### Inclusion criteria

1. Solid gastrointestinal malignancy (esophageal, gastric, small bowel, pancreatic, colon, or rectal cancer).
2. Intravenous 5-FU-based chemotherapy (bolus or continuous infusion).
3. Chest pain occurring during treatment and attributed to 5-FU.
4. Attempted rechallenge with chemotherapy under nitrate and/or calcium channel blocker antianginal treatment. 

#### Exclusion criteria

1. Head and neck malignancies (due to differing treatment protocols and departmental profiles).
2. Oral fluoropyrimidine therapy (e.g., capecitabine).
3. Cardiovascular events during treatment not associated with chest pain (e.g., isolated hypertension, tachycardia, arrhythmia).
4. Antianginal therapy initiated in the absence of 5-FU-related symptoms due to high cardiovascular risk (e.g., known coronary artery disease).
5. Documented acute coronary syndrome caused by confirmed plaque rupture or coronary occlusion.
6. Incomplete medical documentation, particularly missing data regarding chemotherapy rechallenge.

### Patient selection

Among the 835 patients treated for GI malignancies during the study period, 25 patients (2.9%) were identified as having presumed 5-FU-associated coronary vasospasm. During initial screening, 4 cases were excluded for methodological reasons: one due to a head and neck cancer diagnosis, one due to a non-anginal event (isolated tachycardia), one patient receiving prophylactic cardiologic therapy for known three-vessel coronary artery disease in the absence of documented vasospasm, and one case in which chest pain occurred during oral rather than intravenous fluoropyrimidine therapy.

### Statistical analysis

Descriptive statistical analyses were performed using Microsoft Excel. Continuous variables are presented as mean ± SD. Categorical variables are reported as counts and percentages (n, %). Given the small sample size and observational design, no comparative hypothesis testing was performed.

## Results

During the study period between March 1, 2024, and August 1, 2025, a total of 1,908 patients with gastrointestinal malignancies (esophageal, gastric, colorectal, rectal, and pancreatic cancer) were treated at the Day Care Unit of our institution. Of these, 835 patients received intravenous 5-FU-based chemotherapy, and chest pain occurred during treatment in 25 patients (2.9%).

In the affected patient cohort, angina pectoris most commonly developed after the second or third chemotherapy cycle (Fig. 6). Demographic and clinical characteristics of the patients are summarized in Table 1.

**Table 1:** Clinical characteristics of the patient cohort with 5-fluorouracil-induced vasospasm

Number of patients	25
Mean age (years +/-SD)	63.04 +/-10.25
Sex:	n (%)
Female	15 (60)
Male	10 (40)
Tumor localization:	n (%)
Esophagus	3 (12)
Gastric	1 (4)
Pancreas	3 (12)
Colon	18 (72)
Cardiovascular history:	n (%)
Hypertension	17 (68)
Stroke	1 (4)
Atrial fibrillation	2 (8)
Diabetes mellitus	3 (12)
Dyslipidaemia	Not available
Smoking status	Not available

Baseline demographic and clinical data of the 25 patients. Continuous variables are expressed as mean ± standard deviation (SD), while categorical variables are shown as absolute numbers and percentages

Baseline smoking status and lipid profiles were not consistently documented in the electronic medical records and thus could not be reliably analyzed

*Abbreviations*: *5-FU* 5-fluorouracil, *SD* Standard deviation

According to departmental protocol, all patients underwent baseline cardiologic evaluation prior to initiation of chemotherapy, including physical examination, electrocardiography, and transthoracic echocardiography. Management of chest pain occurring during intravenous 5-FU administration followed a predefined institutional protocol.

In cases of chest pain during treatment, chemotherapy was temporarily suspended for 2–3 days, electrocardiography was performed, and cardiac troponin levels were measured if symptoms occurred during hospitalization. After exclusion of acute coronary syndrome, urgent cardiologic reassessment was performed within 2–3 days.

In the absence of new resting electrocardiographic abnormalities (including ST-segment elevation) or regional wall motion abnormalities on echocardiography, chemotherapy was resumed under oral nitrate and calcium channel blocker antianginal treatment, initiated 48 h prior to 5-FU administration and discontinued 48 h after completion. Standard antianginal therapy consisted of isosorbide mononitrate 40 mg or 60 mg once daily, supplemented with amlodipine 2,5–5 mg daily depending on blood pressure and overall cardiovascular status.

If new electrocardiographic abnormalities, including ST-segment elevation, were detected during cardiologic evaluation, urgent coronary angiography within 1–3 days was indicated. In the absence of significant epicardial coronary artery disease, chemotherapy was continued under oral antianginal therapy with continuous monitoring. If coronary stenosis was identified, percutaneous coronary intervention was performed, after which intravenous 5-FU therapy was also continued.

**Fig. 1:**
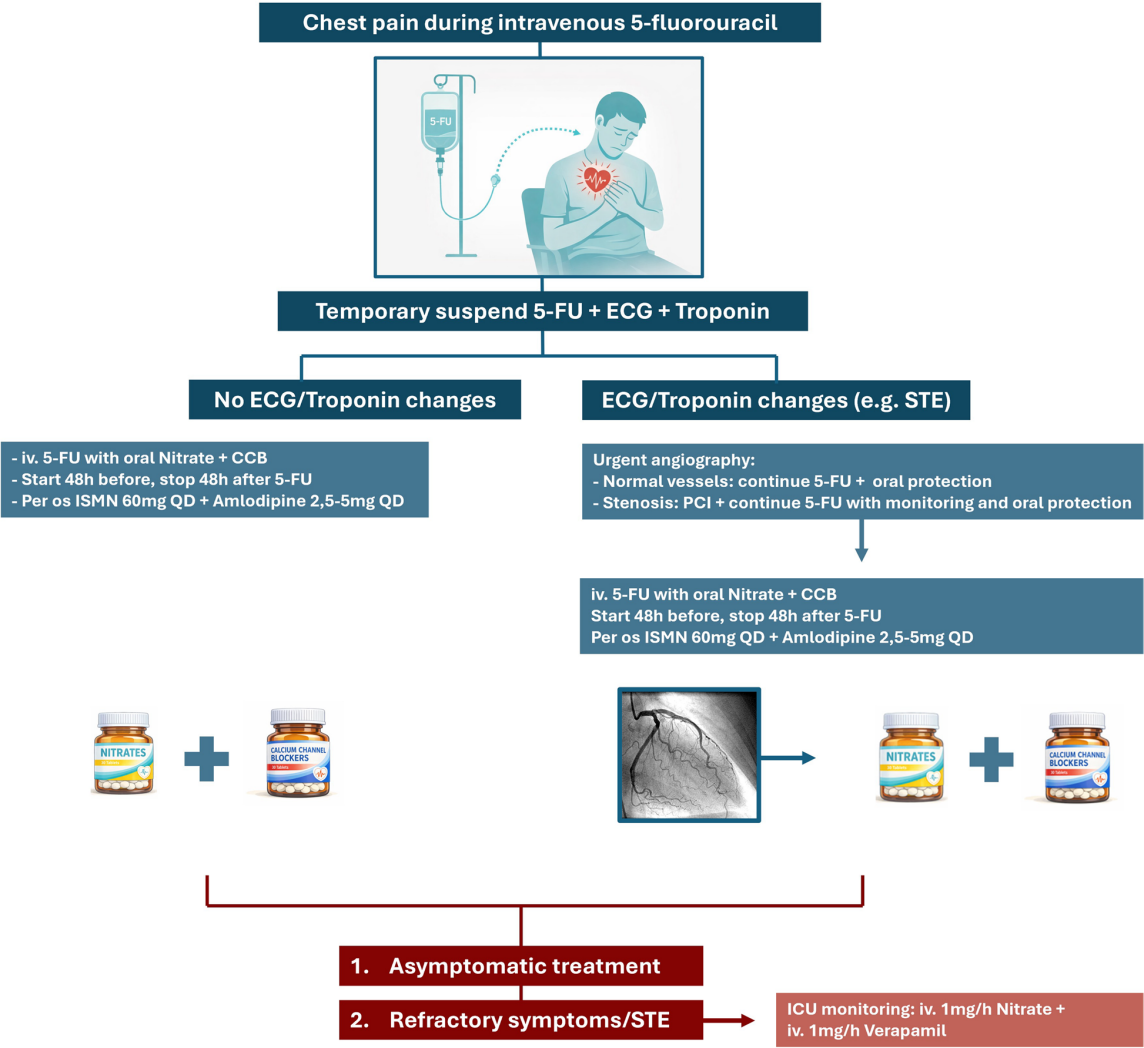
Institutional protocol for chest pain during IV 5-FU treatment. This flowchart outlines the stepwise clinical approach for managing chest pain during intravenous 5-fluorouracil (5-FU) infusion. The purpose was to establish a safe pathway for continuing essential chemotherapy. Upon symptom onset, 5-FU was suspended, followed by urgent cardiological evaluation. Patients without significant coronary artery disease resumed chemotherapy under antianginal protection with nitrates and calcium channel blockers (CCB). This protocol allowed all 25 patients to complete their planned oncological therapy. *Abbreviations: 5-FU: 5-fluorouracil; CCB: calcium channel blocker; ISMN: isosorbide-mononitrate; ECG: electrocardiogram; QD: quaque die (daily); PCI Percutaneous Coronary Intervention; ICU: Intensive Care Unit*

In cases where oral antianginal therapy failed to achieve symptom control and/or persistent ST-segment elevation occurred during intravenous 5-FU administration, chemotherapy was continued in an intensive care setting under continuous monitoring with intravenous nitrate and verapamil therapy. The stepwise institutional management algorithm is illustrated in Fig. 1.

**Fig. 2:**
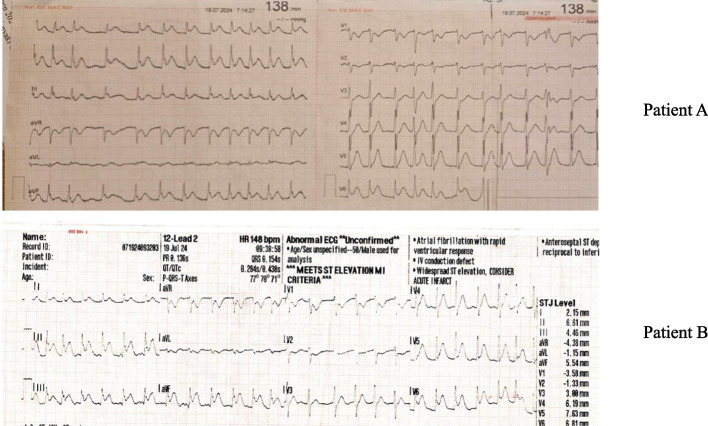
Representative ECG tracings showing 5-fluorouracil-induced ST-elevation. These electrocardiograms document transient ST-elevation (STE) observed in three patients during 5-FU-related chest pain. The objective is to illustrate the clinical presentation mimicking acute coronary syndrome. Despite these abnormalities, imaging confirmed the absence of significant epicardial coronary artery disease, supporting the diagnosis of coronary vasospasm. These findings highlight the importance of urgent cardiological assessment to differentiate vasospasm from plaque rupture. *Abbreviations: ECG: electrocardiogram; STE: ST-elevation; 5-FU: 5-fluorouracil. Symbols: Arrows indicate regions of ST-elevation*

Findings related to cardiologic management and coronary imaging are summarized in Table 2. ST-segment elevation was documented in 3 of the hospitalized patients during episodes of chest pain; representative electrocardiograms are shown in Fig. 2. In patients who experienced angina at home, electrocardiographic documentation was not available, precluding precise determination of the overall incidence of ECG abnormalities.

**Table 2:** Management outcomes of 25 patients affected by 5-FU-induced chest pain, n (%)

New-onset ECG abnormality (STE) during treatment	3 (12)
Coronary imaging	
Coronarography	6 (24)
Coronary CT Angiography	2 (8)
Significant coronary stenosis	0 (0)
Antianginal management for chest pain	
Calcium channel blocker monotherapy (CCB)	2 (8)
Isosorbide mononitrate monotherapy	4 (16)
Combined therapy with CCB and nitrates	19 (76)
Asymptomatic state with antianginal therapy	25 (100)

Results of cardiac evaluations and management outcomes. Coronary imaging (CCTA or invasive angiography) was performed to exclude obstructive coronary artery disease in symptomatic patients. Notably, 100% of the affected patients successfully completed their planned chemotherapy cycles under the antianginal protocol without symptom recurrence

*Abbreviations*: *5-FU* 5-fluorouracil, *CCB* Calcium channel blocker, *CCTA* Coronary CT angiography, *ECG* Electrocardiogram, *STE* ST-segment elevation

**Fig. 3:**
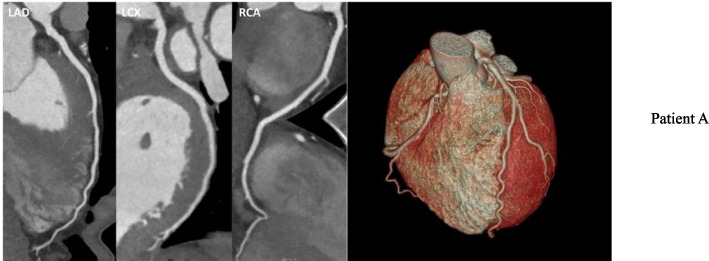
Coronary CT angiography demonstrating normal coronary arteries. This figure presents a representative coronary CT angiography (CCTA) image from a patient experiencing 5-FU-related chest pain (Patient A). Due to the presence of regional hypokinesis on echocardiography, CCTA was utilized to rule out significant coronary stenosis or plaque rupture. Results showed patent epicardial coronary arteries, confirming that anginal symptoms were caused by functional vasospasm rather than structural obstruction. This exclusion is critical for safely initiating antianginal treatment before reintroducing chemotherapy*. Abbreviations: CCTA: coronary CT angiography; 5-FU: 5-fluorouracil*

**Fig. 4:**
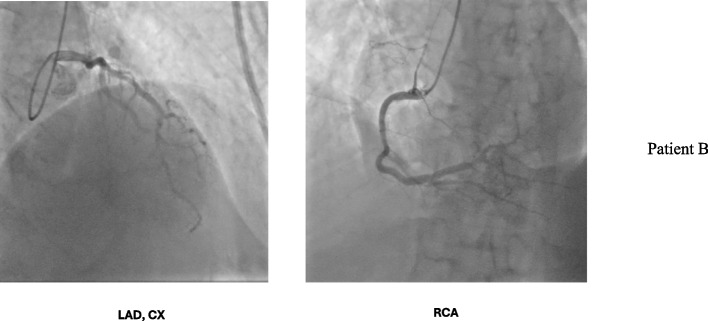
Invasive coronary angiography confirming absence of significant epicardial stenosis. Representative invasive coronary angiography images from the study cohort are shown (Patient B). The purpose was to exclude obstructive coronary artery disease in patients with new ECG abnormalities or persistent symptoms. Results demonstrated no significant atherosclerotic lesions, supporting the diagnosis of 5-FU-induced coronary vasospasm. Such definitive imaging allows for the confident continuation of 5-FU therapy under pharmacological protection. *Abbreviations: 5-FU: 5-fluorouracil; LAD: Left Anterior Descending artery; CX: circumflex artery; RCA: Right Coronary Artery*

High-sensitive cardiac troponin levels were measured in 5 patients following chest pain episodes. Samples were collected within the diagnostic time window, with a second measurement performed if the initial results fell within the 'grey zone'; however, no evidence of myocardial necrosis was found in any case. Transthoracic echocardiography demonstrated preserved left ventricular systolic function in 24 patients, without regional wall motion abnormalities. One patient showed hypokinesis on echocardiography, leading to an elective coronary CT scan. Coronary imaging (invasive coronary angiography or coronary computed tomography angiography) performed after symptom onset did not reveal significant coronary stenosis in any patient. We perform coronary CTA or coronary angiography only for patients who remain symptomatic despite receiving antianginal therapy combined with a negative ECG. In the event of a new-onset ECG abnormality (ST-segment elevation) during chemotherapy, coronary imaging is performed in all cases. Representative coronary imaging studies obtained for angina evaluation are shown in Figs. 3 and 4.

**Fig. 5:**
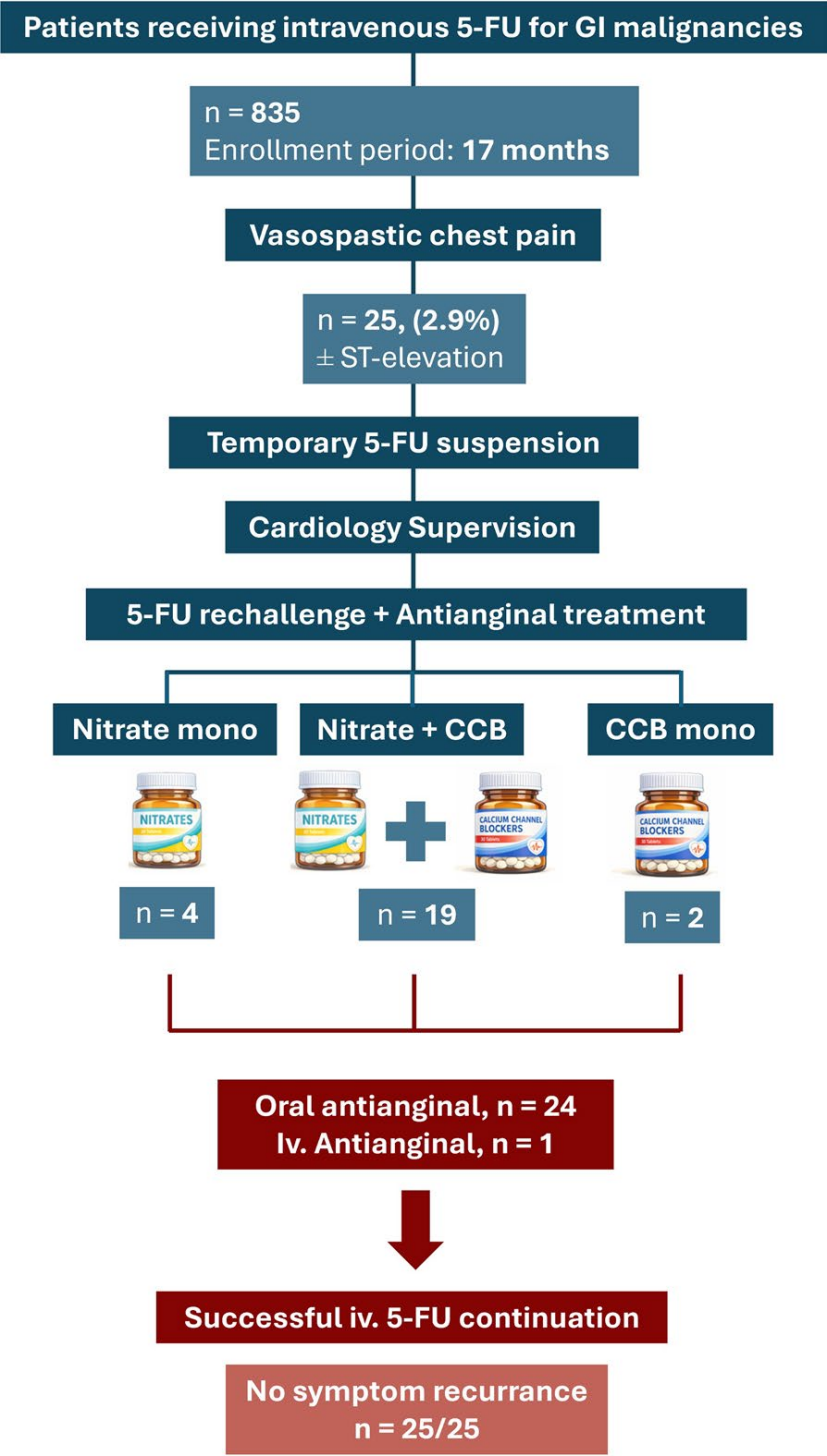
Central illustration: Clinical outcomes of chemotherapy reintroduction under antianginal treatment. This bar chart summarizes the results of continuing 5-FU therapy in 25 patients. The goal was to evaluate the effectiveness of the institutional antianginal protocol. Most patients (19/25) successfully continued treatment without symptom recurrence using combined nitrate and CCB therapy. The results indicate that appropriate medical management effectively prevents the recurrence of coronary vasospasm, ensuring adherence to the oncological treatment plan. *Abbreviations: 5-FU: 5-fluorouracil; GI: gastrointestinal; CCB: calcium channel blocker*

Intravenous 5-FU therapy was successfully continued under oral nitrate and calcium channel blocker antianginal treatment in 24 patients without recurring chest complaint. One patient required intravenous antianginal therapy in an intensive care setting due to persistent chest pain; symptom resolution was achieved, allowing continuation of chemotherapy. Importantly, none of the 25 patients required permanent discontinuation of 5-FU-based oncologic therapy. Clinical experience with successful cardiologic management during chemotherapy rechallenge is summarised in Fig. 5.

If a patient experiences chest discomfort during 5-FU administration, the subsequent cycle is administered in an inpatient setting. If the patient becomes completely asymptomatic under nitrate and CCB therapy, further treatment can be continued on an outpatient basis.

**Fig. 6:**
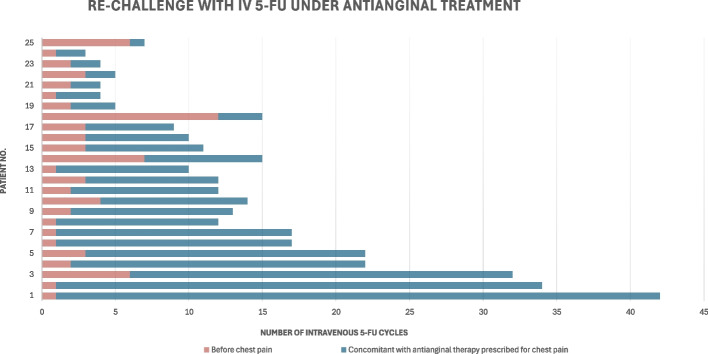
Oncological relevance of continuing chemotherapy despite vasospasm. This graph illustrates the oncological impact of the antianginal protocol. Timeline of 5-FU administration for each patient (*N* = 25) until November 18, 2025. Red segments represent completed 5-fluorouracil cycles prior to cardiotoxicity; blue segments indicate subsequent cycles successfully administered under a standardized antianginal protocol. This approach prevented premature treatment termination, allowing for the continuation of primary oncologic therapy despite 5-FU-induced vasospasm. Such multidisciplinary management is essential for optimizing cancer care. *Abbreviations: 5-FU: 5-fluorouracil*

The oncologic relevance of chemotherapy rechallenge following coronary vasospasm is illustrated in Fig. 6. The figure shows the number of 5-FU-based chemotherapy cycles patients would have missed (blue line) compared with the actual treatment received (red line) had therapy been permanently discontinued due to chest pain in the absence of disease progression and replaced with a typically less effective alternative regimen. At the end of follow-up (November 18, 2025), 11 patients were still receiving intravenous 5-FU-based therapy, 6 patients had completed adjuvant chemotherapy, and 8 patients required a change in therapy due to disease progression.

## Discussion

Appropriate management of chest pain caused by presumed coronary vasospasm associated with 5-fluorouracil (5-FU) therapy enables continuation of chemotherapy and prevents premature treatment modification in the absence of disease progression. In a large U.S. study including 6,606 patients, survival data demonstrated that among the 115 patients who developed coronary vasospasm, rechallenge with 5-FU following appropriate antianginal therapy significantly improved both progression-free and overall survival. Median overall survival was 47 months in patients who resumed 5-FU, compared with 18 months in those in whom the causative chemotherapy was permanently discontinued [9].

A relevant methodological consideration is that although our institution has established guidelines for the management of presumed 5-FU-induced vasospasm, the retrospective nature of the present study precluded detailed description of protocol elements within the Methods section. Instead, treatment steps were reported in the Results section, allowing the presentation of individualised therapeutic decisions implemented in real-world clinical practice rather than a purely theoretical algorithm, along with their observed effectiveness.

Over a 17-month period, 835 patients with gastrointestinal malignancies received intravenous 5-FU therapy at our Institution. Chest pain occurred in 25 patients (2.9%). Under the protection of the institutional antianginal protocol, every affected patient successfully continued their planned chemotherapeutic regimen. By comparison, the largest single-center U.S. cohort study reported that among 6,060 predominantly gastrointestinal cancer patients treated with 5-FU over 19 years, vasospasm occurred in 1.74% of patients, and chemotherapy could be resumed in 78 patients (67.8%) following initiation of antianginal therapy [9]. Although no recurrent cardiotoxicity was observed in our cohort, this high completion rate must be interpreted with caution given our limited sample size compared to the 67.8% success rate reported by Zafar et al. This variance is likely secondary to patient selection and specific institutional protocols rather than a definitive clinical superiority. Consequently, larger prospective studies are warranted to fully evaluate the efficacy of our protocol. In another large cohort study, 4,019 patients received 5-FU-based chemotherapy over 10 years, with coronary vasospasm documented in 87 patients (2.16%) [1]. Several studies have reported recurrence of chest pain in up to 90% of cases when 5-FU is rechallenged without antianginal protection following vasospasm [4].

The mean age at symptom onset in our cohort was 63 years, compared with 58 years in another study of 87 affected patients [1]. This difference likely reflects the epidemiology of gastrointestinal malignancies. Of the 25 patients in our cohort, 15 (60%) were women and 10 (40%) were men, whereas in the previously cited U.S. study, women accounted for 42.5% of cases [1]. Given the limited sample size and the presence of multiple non-oncologic comorbidities, no meaningful conclusions regarding sex-specific risk can be drawn.

Regarding cardiovascular comorbidities, 68% of patients had known hypertension and 12% had diabetes mellitus prior to initiation of chemotherapy. Smaller proportions had a history of atrial fibrillation, stroke, or peripheral arterial disease. In the international literature, one large cohort study reported a prevalence of hypertension of 46% (40/87) among patients who developed vasospasm, compared with 57.5% (100/174) among those who did not [9]. One potential explanation is that calcium channel blockers—commonly used for hypertension—are also first-line agents for the treatment of coronary vasospasm. Based on available data, the relationship between baseline cardiovascular risk and the development of coronary vasospasm during intravenous 5-FU therapy remains controversial [4, 9]. In our study, the small cohort size precludes drawing definitive conclusions regarding the predictive role of baseline cardiovascular comorbidities. Our study also highlights a gap in routine clinical documentation: cardiovascular risk factors such as smoking status and lipid profiles were often under-reported in oncology outpatient records. This underscores the need for more integrated cardio-oncology electronic health records to stratify patient risk better.

The 2022 European Society of Cardiology Guidelines on Cardio-Oncology emphasize the importance of cardiovascular risk assessment prior to initiation of fluoropyrimidine-based chemotherapy, including baseline electrocardiography and echocardiography in higher-risk patients [14]. At our institution, all patients undergo baseline cardiologic evaluation—including ECG and transthoracic echocardiography—prior to chemotherapy initiation to enable informed oncologic treatment planning, particularly when potentially cardiotoxic agents are used. Cardiac troponin may serve as a useful prognostic biomarker for detecting 5-FU-associated cardiotoxicity. In our study, routine troponin monitoring at symptom onset was not feasible in most cases, as chest pain frequently occurred at home and was reported retrospectively. Troponin levels were measured in 5 patients during chest pain episodes, with no evidence of elevation. In contrast, the previously cited U.S. cohort study reported elevated conventional or high-sensitivity troponin levels in 69.4% of affected patients at symptom onset [1].

In our institution, the Troponin T assay is available. It is indeed true that different institutions use different troponin assays, reagents, and reference ranges, which complicates standardised evaluation. However, from a cardiological perspective, the different troponin types do not affect the uniformity of the treatment protocol.

A standardized protocol for the management of chest pain due to coronary vasospasm is essential for minimizing adverse effects and enabling continuation of oncologic therapy. Following multidisciplinary cardio-oncology consultation, treatment may be safely resumed under close cardiologic monitoring with long-acting nitrate and calcium channel blocker therapy. In most cases, we recommend a 60 mg dose of nitrate. However, if the systolic blood pressure is below 100 mmHg, a 40 mg dose is administered. If the patient is already taking their own antihypertensive medication, we recommend adding 5 mg of amlodipine. If they are on a combination antihypertensive therapy that already includes a CCB, no additional medication is introduced. If the patient is not taking any antihypertensive medication, we recommend 5 mg of amlodipine for systolic blood pressure values above 100 mmHg, and 2,5 mg below this threshold. Rechallenge strategies may include fluoropyrimidine dose reduction, concomitant anti-ischemic therapy, avoidance of physical exertion, and aggressive control of lipid levels, hypertension, and other cardiovascular risk factors [14].

In a U.S. study of 78 patients, prophylaxis for recurrent chest pain during intravenous 5-FU consisted of nitrate monotherapy in 19 patients, calcium channel blocker monotherapy in 15 patients, and combination therapy in 44 patients. No significant difference in recurrence rates was observed between monotherapy and combination therapy [9]. Increasing numbers of international studies have also reported successful rechallenges with 5-FU following chest pain using nitrate and/or calcium channel blocker therapy [4, 15–17]. In our cohort, all 25 patients were able to continue oncologic treatment with appropriate cardiologic management. Combination antianginal therapy was used in 19 patients, nitrate monotherapy in 4 patients, and calcium channel blocker monotherapy in 2 patients. The most common adverse effects requiring therapy modification were nitrate-induced headache and calcium channel blocker-associated hypotension or bradycardia. Consequently, routine prophylactic antianginal therapy is not administered to all patients receiving intravenous 5-FU in the absence of symptoms, in order to avoid unnecessary adverse effects.

In the broader context of managing fluoropyrimidine-induced cardiotoxicity, alternative treatment strategies deserve consideration. Although S-1 (tegafur-gimeracil-oteracil) is not routinely available in Hungary and thus not part of our standard practice, published evidence suggests that switching to S-1 carries a substantially lower risk of recurrent cardiotoxicity than 5-FU or capecitabine. Furthermore, a 2022 meta-analysis confirmed comparable oncological efficacy regarding progression-free and overall survival [18]. Consequently, ESMO-based guidance and expert consensus now consider S-1 a reasonable alternative for these high-risk patients, despite the lack of prospective randomized trials [19].

### Study limitations

The principal limitation of our study is the relatively small sample size, primarily resulting from the exclusion of patients with head and neck malignancies and those treated with oral fluoropyrimidines. Nevertheless, a review of the international literature indicates that, aside from a single-centre cohort study conducted at Boston University [9], current evidence on the management of 5-FU-induced anginal symptoms is largely derived from case reports and small case series. A multicenter study encompassing both intravenous and oral fluoropyrimidine therapies across oncologic diagnoses would substantially enhance the evidence base and inform clinical practice.

Furthermore, in our study, the small cohort size precludes drawing definitive conclusions regarding the relationship between coronary vasospasm and baseline cardiovascular risk factors or demographic characteristics.

The retrospective nature of case identification via pharmacy records for the specific protocol medication (nitrates) may have led to an underestimation of cases managed outside this protocol or of those in which 5-FU was permanently discontinued immediately. Future prospective registries are needed to confirm these findings.

An additional limitation was the absence of electrocardiographic and troponin measurements in several cases in which chest pain occurred outside the hospital setting, as patients did not seek immediate medical attention. Therefore, these events are best characterized as presumed 5-FU-induced vasospasm. This highlights the importance of patient education at the initiation of oncologic therapy and emphasises the need for prompt medical evaluation in the event of chest pain during fluoropyrimidine treatment. International evidence-based guidelines are needed to standardize antianginal protocols for 5-FU-induced cardiotoxicity.

## Conclusion

In summary, the successful management of cardiotoxic adverse effects associated with oncologic therapies highlights the critical importance of integration between oncology and cardiology. Based on our findings, and in comparison with the international literature, coronary vasospasm-related chest pain associated with intravenous 5-fluorouracil therapy represents a rare but clinically meaningful complication that requires prompt recognition and targeted treatment. According to our institutional experience, initiation of antianginal therapy with nitrates and calcium channel blockers allowed safe continuation of oncologic treatment in all affected patients, which may facilitate the completion of planned chemotherapy. Due to the limited cohort size, no definitive links between coronary vasospasm development and baseline cardiovascular risk or demographic characteristics could be established in this study.

While these findings highlight a potential pathway to maintain oncological dose intensity, further prospective studies are required to assess the clinical presentation, cardiovascular risks, demographic characteristics, management strategies and the long-term impact on survival and clinical outcomes of patients with 5-FU-associated coronary vasospasm and to facilitate the development of standardized management protocols.

Alternative fluoropyrimidines with a lower propensity to induce coronary vasospasm (e.g., S-1/tegafur, trifluridine/tipiracil) are not currently recommended as first-line therapies in international guidelines, and their availability remains limited in Hungary. However, while not utilized upfront, their administration should be strongly considered as a valuable alternative strategy for patients who develop fluoropyrimidine-induced cardiotoxicity. Moreover, switching agents prematurely may limit future therapeutic options as the disease progresses.

### Perspectives

#### Competencies in medical knowledge

The diagnosis of chest pain due to coronary vasospasm during 5-fluorouracil (5-FU) treatment should not automatically lead to the definitive cessation of necessary oncological therapy. Implementing a structured, institution-specific protocol ensures the safe and successful rechallenge of 5-FU. Combined anti-anginal therapy, specifically oral nitrates and calcium channel blockers (CCBs), is highly effective in managing presumed 5-FU-induced vasospasm, enabling patients to safely continue chemotherapy without symptoms.

#### Translational outlook

Further research is needed through prospective, multicenter studies to validate this anti-anginal management protocol in larger patient cohorts, including those receiving oral fluoropyrimidines. Clinical efforts are required to improve patient education, focusing on immediate symptom recognition and reporting, as late presentation hinders timely ECG and biomarker assessment. Research should identify patients at higher cardiovascular risk to guide prophylactic strategies and help standardize official international guidelines for anti-anginal treatment protocols.

## Data Availability

No datasets were generated or analysed during the current study.

## Abbreviations

5-FU: 5-Fluorouracil
STE: ST-elevation
ECG: Electrocardiogram
CCB: Calcium Channel Blocker
GI: Gastrointestinal
PCI: Percutaneous Coronary Intervention
iv.: Intravenous
per os: Orally administered
SD: Standard Deviation
OS: Overall Survival
CCTA: Coronary Computed Tomography Angiography
hs-cTn: High-sensitivity cardiac troponin
CAD: Coronary Artery Disease
